# NHANES cross sectional study of aspirin and fractures in the elderly

**DOI:** 10.1038/s41598-023-29029-6

**Published:** 2023-02-01

**Authors:** Sarya Swed, Amro A. El-Sakka, Yasmeen Abouainain, Ka Yiu Lee, Bisher Sawaf, Mhd Kutaiba Albuni, Elias Battikh, Eman Mohammed sharif Ahmad, Nashaat Kamal Hamdy Elkalagi, Kirellos Said Abbas, Wael Hafez, Amine Rakab

**Affiliations:** 1 Faculty of Medicine, Aleppo University, Aleppo, 22743 Syria; 2 Faculty of Medicine, Suez Canal University, Ismailia, Egypt; 3 Faculty of Medicine, University of Jordan, Amman, Jordan; 4 Department of Health Sciences, Swedish Winter Sports Research Centre, Mid Sweden University, Östersund, Sweden; 5 Department of Internal Medicine, Hamad Medical Corporation, Doha, Qatar; 6 Nile Valley University, Atbra, Sudan; 7 Internal Medicine and Tropical Medicine at Faculty of Medicine Al Arish University, Al Arish, Egypt; 8 Faculty of Medicine, Alexandria University, Alexandria, Egypt; 9 NMC Royal Hospital, 16th Street, Khalifa City, Abu Dhabi UAE; 10 Medical Research Division, Department of Internal Medicine, The National Research Centre, Cairo, Egypt; 11 Assistant Professor of Clinical Medicine, Medicine, Weill Cornell Medical College, Ar Rayyān, Qatar

**Keywords:** Health care, Medical research, Risk factors

## Abstract

Bone fractures are a global public health concern, yet no thorough investigation of low-dose aspirin usage to prevent fractures in the elderly has been conducted. Many interventional human and animal studies have tried to detect the correct role of low-dose aspirin on fractures in elderly persons. The literature doesn't consist of a retrospective observational study that includes a large number of older individuals and evaluates the accurate effect of aspirin on the fractures post falling from low heights. This cross-sectional includes 7132 elderly persons and aimed to detect if there was a link between taking low-dose aspirin to prevent fractures in the elderly. Data was extracted from the National Health and Nutrition Examination Survey (NHANES) database for 2017–2020 and 2013–2014. Demographic and examination data were collected during in-home interviews and study visits to a mobile examination center. Standardized questionnaires were used to collect information such as age, gender, race, educational level, and family income-to-poverty ratio. Body mass index (BMI), weight, standing height, upper leg length, upper arm length, arm circumference, and wrist circumference were all measured during the examination. The study examined 8127 patients, with 7132 elderly patients suitable for data analysis. The odds ratio of fractures due to a fall from standing height or less was 0.963 (95 percent confidence interval 0.08–1.149) in low-dose aspirin users, while having parents with osteoporosis had a related risk of 1.23. (95 percent confidence interval 0.81–1.8). The total number of fractures was 1295; with hip fractures constituting up to 13.82%, wrist fractures of 66.56%, and spine fractures of 19.61%. There was no significant difference in femur and spine bone mineral density (BMD) in the two groups (use low dose aspirin and don't use). Females had a 5.6 times greater fracture risk related to a fall from standing height or less (1 time or more) than males (P-value < 0.001). Furthermore, taking aspirin had no effect on the occurrence of fractures from standing height or less in older people (P-value = 0.468). In addition, the logistic regression after performing the propensity matching score confirmed that there was no impact of taking aspirin on the occurrence of fractures (P-value > 0.05). This cross-sectional study reveals that taking low-dose aspirin to prevent fractures in the elderly is statistically insignificant. However, fractures are more common in older persons, especially in older women; thus, more widespread injury prevention initiatives and access to osteoporosis prevention and diagnosis for older people should improve to minimize the overall burden.

## Introduction

Fractures are a significant public health problem, and their incidence is steadily increasing worldwide. In 2019, it was estimated that there were more than 178 million fractures worldwide, with the incidence remarkably increasing in older age groups, mainly due to osteoporosis^1,2^. By 2025, it is estimated that the incidence of fractures due to osteoporosis among Americans will surpass 3 million cases, with an economic burden exceeding $25 billion annually^3^. Increased life expectancy can explain the rising number of osteoporotic instances^4^, raising an alarming call for action. Osteoporotic fractures significantly burden patients and their families, considering their high morbidity and mortality^5,6^. They impair patients' quality of life and have a harmful impact on their physical and mental well-being^7^.

Osteoporosis is a long-standing illness characterized by reduced bone density that predisposes to fractures at relatively low levels of trauma^8^. It is usually associated with older age groups, as bone loss is accelerated in postmenopausal women, and in men, reduced steroids production also plays a role^9^. Furthermore, several studies suggested that inflammatory mediators, like tumor necrosis factor (TNF)-α, interleukin (IL)-6, and C reactive protein (CRP), can enhance osteoclastic activity and accelerate bone loss^10–12^. Prostaglandins have also been found to have bone resorptive effects in the long term^13,14^. As a cyclooxygenase (COX) inhibitor, Aspirin reduces inflammatory cytokines and adjusts the prostaglandins' effects on bone, which may decelerate bone resorption.

Several studies investigated the effect of aspirin on bone resorption and remodeling. Some studies found aspirin to preserve bone mineral density (BMD)^14–19^, whereas others didn't find such an association^20–22^. Considering the conflicting nature of the available results and the gravity of the topic, we sought to investigate this relationship further by analyzing data from thousands of patients through the National Health and Nutrition Examination Survey (NHANES) database.

## Materials and methods

### Study population

Data for this study were obtained from the NHANES database. NHANES was developed to evaluate the health and nutritional status of the US population. It is conducted by the National Center for Health Statistics within the US Centers for Disease Control and Prevention. Data collection in our study targeted the records between 2013 and 2014 and between 2017 and 2020, using a complex, multi-stage, hierarchical, clustered probability sample design to select a representative sample of civilians rather than a simple random sample based on the US population^23^.

**Figure 1:**
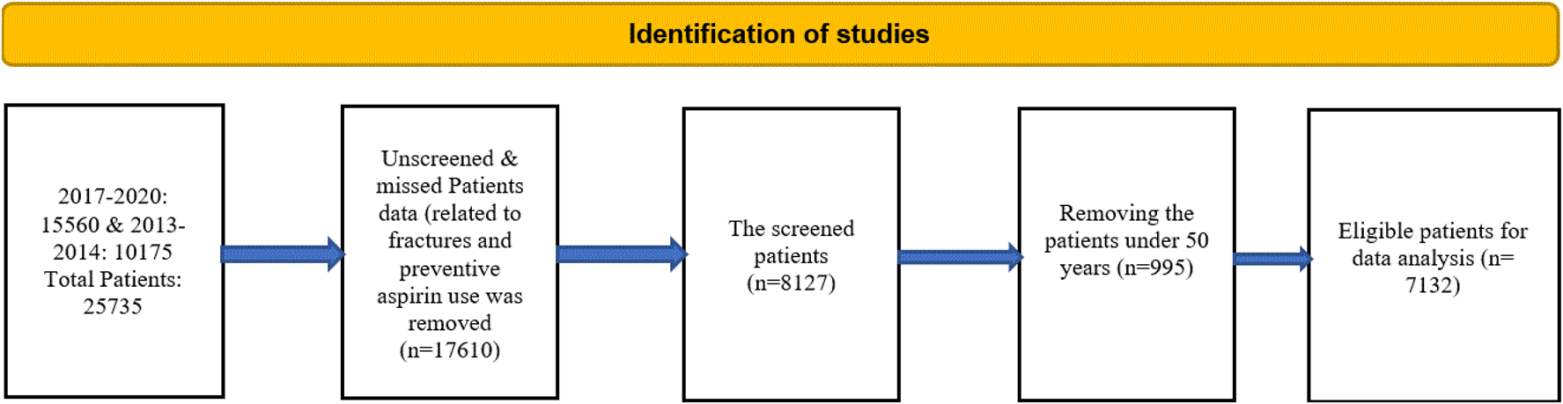
Flow chart of the included patients from the National Health and Nutrition Examination Survey (NHANES) database within 2013–2104 and 2017–2020.

The study population was adults over 50 years of age. A total number of participants over the study period were 25,737 people. Upon assessing records with unscreened or missed patients’ data (related to fractures and preventive aspirin use), 17,610 records were excluded. After further exclusion of patients under 50 years of age (n = 995), 7132 patients were eligible for analysis (Fig. 1). NHANES was approved by the National Center for Health Statistics research ethics review board (https://wwwn.cdc.gov/Nchs/Nhanes/). Written informed consent was obtained from all participants. All methods were performed in accordance with the relevant guidelines and regulations.

### Evaluation of exposures

The questions on the prophylactic usage of aspirin were inquired about by trained interviewers using the Computer-Assisted Personal Interview (CAPI) technology in the participants' homes. The Dietary Supplements and Prescription Medicine portion of the Sample Person Questionnaire, which gathers information about dietary supplements, nonprescription antacids, and prescription medication, included the administration of these questions. To minimize data entering mistakes, the CAPI system is developed with built-in consistency checks. Additionally, CAPI makes use of online help screens to assist interviewers in explaining important terminology included in the questionnaire.

### Outcomes

The frequencies of the fractures from standing height or less (1 time or more), indicating osteoporosis, were merged with the other variables, such as demographic characteristics, body measures and the data of low dose aspirin usage. This variable was categorized into the following groups: hip fractures, wrist fractures, and spine fractures. This was based on self-reporting by the participants.

### Covariates

Demographic and examination data were collected during in-home interviews and study visits at a mobile examination center. Data about age, gender, race, educational level, and the ratio of family income to poverty were collected via standardized questionnaires. At the same time, examination data covered body mass index (BMI), weight, standing height, upper leg length, upper arm length, arm circumference, and wrist circumference.

### Statistical analysis

We used Student’s t-test, and a Chi-square test of proportions was used to compare whether the means (of continuous variables) or proportions (of categorical variables) of demographic and examination characteristics were significantly different between aspirin users and non-users. A P-value of 0.05 was used as a marker of statistical significance. MedCalc estimated odds ratio to examine the relationship between aspirin use and different types of fractures. All analyses were performed using SPSS 26.0 (SPSS, Inc., Chicago, IL, USA). In addition, binary logistic regression was conducted to explore the predicted correlation between the fractures due to falls from standing height or below and other variables. As well, we used STATA 15 software to perform propensity score matching to create a to matched groups; treated with low-dose aspirin (n = 748) and not treated (n = 1062).

## Results

### Sociodemographic-baseline characteristics of the study sample

Almost of the sample study had no prior fractures (86.3%). The average age among the persons who had one fracture at least in the past (65.82 ± 9.36) is larger more than the persons who had no (64.58 ± 9.20). The BMI was larger among females (30.49 ± 7.8) and the persons who had one fracture at least (29.83 ± 6.88) than males (29 ± 5.9) and the persons who had no fracture (29.75 ± 7.04). Furthermore, the total femur BMD, total spine BMD among the persons who had no fractures are larger than the persons who had one prior fracture at least in the past (Table 1).

**Table 1:** Baseline characteristics of the study sample.

Variables	Category	Gender		Fracture	
		Male: 3484(48.9%)	Female: 3648(51.1%)	Yes: 597(13.7%)	No: 6175(86.3%)
		Frequency (Percentage) /Mean ± Standard deviation	Frequency (Percentage) /Mean ± Standard deviation	Frequency (Percentage) /Mean ± Standard deviation	Frequency (Percentage) /Mean ± Standard deviation
Age		64.89 ± 9.1	64.60 ± 9.2	65.82 ± 9.36	64.58 ± 9.20
Race/Hispanic origin	Mexican American	379(5.3%)	361(5.1%)	72(1.0%)	668(9.4%)
	Other Hispanic	317(4.4%)	375(5.3%)	87(1.2%)	605(8.5%)
	Non-Hispanic White	1443(20.2%)	1443(20.2%)	548(7.7%)	2411(33.8%)
	Non-Hispanic Black	885(12.4%)	889(12.5%)	177(2.5%)	1597(22.4%)
	Other Race – Including (Multi-Racial)	460(6.4%)	507(7.1%)	91(1.3%)	876(12.3%)
Country of Birth	Born in 50 US states or Washington, DC	2518(35.3%)	2587(36.3%)	808(11.3%)	4297(60.2%)
	Born in Mexico	966(13.5%)	1057(14.8%)	167(2.3%)	1856(26.0%)
	Refused	0	4(0.1%)	0(0%)	4(0.1%)
Educational level	Less than 9th grade	385(5.4%)	357(5.0%)	87(1.2%)	655(9.2%)
	9-11th grade (Includes 12th grade with no diploma)	455(6.4%)	411(5.8%)	103(1.4%)	763(10.7%)
	High school graduate/GED or equivalent	842(11.8%)	910(12.8%)	248(3.5%)	1504(21.1%)
	Some college or AA degree	940(13.2%)	1159(16.3%)	340(4.8%)	1759(24.7%)
	College graduate or above	855(12.0%)	806(11.3%)	197(2.8%)	1464(20.5%)
	Refused	0(0.0%)	1(0.0%)	0(0.0%)	1(0.0%)
	Don't know	7(0.1%)	4(0.4%)	0(0.0%)	11(0.2%)
Ratio of family income to poverty*	2.5 or below 2.6 and above	1632(26.3%) 1795(28.9%)	1405(22.7%) 1371(22.1%)	500(8.1%) 365(5.9%)	2927(47.2%) 2411(38.9%)
Body mass index		29 ± 5.9	30.49 ± 7.8	29.83 ± 6.88	29.75 ± 7.04
Total protein (g/dL) **		7.09 ± 0.46	7.06 ± 0.48	70.2407 ± 4.79660	70.88 ± 4.74
cigarettes smoked per day***		18.71 ± 16.19	14.52 ± 14.07	19.62 ± 16.5	16.68 ± 15.3
Triglycerides (mg/dL) **		155.82 ± 127.8	143.58 ± 84.25	114.68 ± 62.9	118.26 ± 95.8
Cholesterol (mmol/L) **		4.67 ± 1.1	5.15 ± 1.09	188.8 ± 43.3	190.4843.5
Total spine BMD****		1.053 ± 0.17	0.95 ± 0.16	0.974 ± 0.17	1.0004 ± 0.17
Total femur BMD*****		0.99 ± 0.14	0.85 ± 0.14	0.8967 ± 0.17	0.9325 ± 0.15
Creatinine (mg/dL) **		1.1 ± 0.7	0.84 ± 0.48	1.0059 ± 0.7	0.9728 ± 0.60
Phosphorus (mg/dL) **		3.5 ± 0.5	3.78 ± 0.54	3.67 ± 0.61	3.64 ± 0.5
Osmolality (mmol/Kg) **		282.3 ± 5.93	282.1 ± 85.7	281.9 ± 6.2	282.2 ± 5.7
Total calcium (mg/dL) **		9.32 ± 0.38	9.40 ± 0.40	2.33 ± 0.10	2.34 ± 0.09
Bicarbonate (mmol/L) **		25.7 ± 2.4	25.78 ± 2.36	25.67 ± 2.4	25.77 ± 2.41
Weight (kg)		86.86 ± 20.26	76.96 ± 21.19	82.9 ± 22.087	81.61 ± 21.20
Standing Height (cm)		172.72 ± 7.67	158.68 ± 6.92	166.36 ± 10.58	165.41 ± 10.04
Upper Leg Length (cm)^&^		40.33 ± 3.12	35.99 ± 3.04	38.42 ± 3.89	38.09 ± 3.7
Upper Arm Length (cm)^&&^		39.11 ± 2.45	35.90 ± 2.37	37.80 ± 2.98	37.43 ± 2.88
Arm Circumference (cm)^&&&^		33.53 ± 4.52	32.64 ± 5.46	33.0324 ± 5.29	33.09 ± 5
Wrist Circumference (cm)^&&&&^		104.45 ± 15.10	100.95 ± 15.93	104.01 ± 16.07	102.4 ± 15.55

*Missing data: 13%, **Missing data: 6.3%, ***Missing data: 68.8%, ****Missing data: 53.2%, *****Missing data: 15.9%, &Missing data: 5.8%, &&Missing data: 4.1%, &&&Missing data: 4.2%, &&&&Missing data: 5.3%.

The average and standard deviation age was 62.9 ± 9.02 for non-aspirin users, and 67.5 ± 8.8 for low-dose aspirin users. Non-aspirin users made up more females than males, while low-dose aspirin users made up more males than females. Non-aspirin users had a family income-to-poverty ratio of 2.64 ± 1.6, whereas low-dose aspirin users had a ratio of 2.61 ± 1.5. The percentage of persons who take aspirin is 38.6%; 35% were by consultation of a doctor and 3.6% were a decision taken by oneself (Table 2).

**Table 2:** Weighted Characteristics of Study Sample Non-aspirin users and low-dose aspirin users.

Variables	Category	Low dose aspirin use		P-value
		No (n = 4381)	Yes (n = 2751)	
Age		62.9 ± 9.02	67.5 ± 8.8	< 0.001
Gender	Male	2009 (28.2%)	1475 (20.7%)	< 0.001
	Female	2372 (33.3%)	1276 (17.9%)	
Race/Hispanic origin	Mexican American	497 (7.0%)	243 (3.4%)	< 0.001
	Other Hispanic	481 (6.7%)	211 (3.0%)	
	Non-Hispanic White	1668 (23.4%)	1291 (18.1%)	
	Non-Hispanic Black	1066 (14.9%)	708 (9.9%)	
	Other Race—Including Multi-Racial	669 (9.4%)	298 (4.2%)	
Educational level	Less than 9th grade 9-11th grade (Includes 12th grade with no diploma) High school graduate/GED or equivalent Some college or AA degree College graduate or above Refused Don't know	506 (7.1%) 1018 (14.3%) 1287 (18.0%) 1072 (15.0%) 0 (0.0%) 5 (0.1%)	360 (5.0%) 734 (10.3%) 812 (11.4%) 589 (8.3%) 1 (0.0%) 6 (0.1%)	< 0.001
Ratio of family income to poverty		2.64 ± 1.6	2.61 ± 1.5	0.65
Body mass index		29.43 ± 7.1	30.28 ± 6.9	0.48
Weight (kg)		80.50 ± 21.2	83.86 ± 21.3	0.83
Standing Height (cm)		165.15 ± 10.1	166.15 ± 10.0	0.201
Upper Leg Length (cm)		38.09 ± 3.7	38.20 ± 3.8	0.21
Upper Arm Length (cm)		37.28 ± 2.9	37.81 ± 2.8	0.57
Arm Circumference (cm)		32.87 ± 5.0	33.41 ± 5.02	0.015
Wrist Circumference (cm)		101.26 ± 15.5	104.95 ± 15.4	< 0.001
Total calcium (mg/dL)		9.35 ± 0.39	9.41 ± 0.375	< 0.001
Creatinine (mg/dL)		0.93 ± 0.62	0.99 ± 0.22	< 0.001
Phosphorus (mg/dL)		3.66 ± 0.57	3.6 ± 0.57	< 0.001
Total spine BMD		0.9 ± 0.17	1 ± 0.18	< 0.001
Total femur BMD		0.92 ± 0.155	0.93 ± 0.15	< 0.001

(Mean ± SD for continuous variables: P value was calculated by weighted linear regression model. Percent for categorical variables: P value was calculated by weighted chi-square test).

### Aspirin using, fracture from falling from standing height or less, and parents osteoporosis

**Figure 2:**
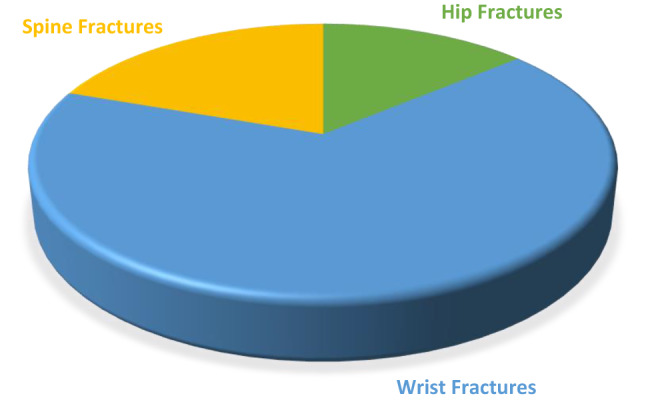
Pie chart figure of the overall fractures (2013–2014 and 2017–2020).

The odds ratio of a hip fracture caused by a fall from a standing height (once or multiple times) is 1.015. The odds ratio of having an in the wrist because of a fall from a standing height (once or multiple times) is 1.02. The odds ratio of a spine fracture caused by a fall from a standing height (once or multiple times) is 0.916. The odds ratio of having a fracture in general due to a fall from a standing height (once or more) is 0.963, and the odds ratio of having parents with osteoporosis is 1.23 (Table 3). Figure 2 revealed that the overall number of fractures was 1295; with hip fractures constituting up to 13.82%, wrist fractures of 66.56%, and spine fractures of 19.61% (Table 3).

**Table 3:** Estimating odds ratio of preventive low dose aspirin use and having the parent’s osteoporosis with occurrence of fractures due to a fall from standing height or less.

Statement	Non-User aspirin	User aspirin	Parents had no osteoporosis	Parents had osteoporosis
Fracture in the hip due to a fall from standing height or less (1time or more)				
Yes	31	19	–	–
No	4350	2732		
Odds ratio (95%, CI: Range)	1.015 (95%; CI:0.71–0.145)		–	
Fracture in wrist due to a fall from standing height or less1time or more)				
Yes	77	51	–	–
No	4304	2700		
Odds ratio (95%, CI: Range)	1.02 (95%; CI:0.88–1.18)		–	
Fracture in spine due to a fall from standing height or less (1time or more)				
Yes	11	8	–	–
No	4370	2743		
Odds ratio (95%, CI: Range)	0.916 (95%; CI: 0.05–1.5)		–	
Fracture due to a fall from standing height or less (1 time or more)				
Yes	114	76	170	20
No	4267	2675	6042	900
Odds ratio (95%, CI: Range)	0.963 (95%; CI: 0.08–1.149)		1.23 (95%; CI:0.81–1.8)

### Multivariable regression between occurrence of fracture from falling from standing height or less (1 time or more) and other variables

#### Before propensity matching score performance

The logistic regression model was statistically significant, X2(6) = 52.49, P-value < 0.001. Only one was statistically significant of the five predictor variables: gender. Females had a 5.6 times higher fracture risk due to a fall from standing height or less (1 time or more) than males. In addition, taking aspirin was not associated with decreasing or increasing the occurrence of fractures from standing height or less in elderly persons (P-value = 0.468) (Table 4).

**Table 4:** Binomial logistic regression between the occurrence of fracture due to a fall from standing height or less (1 time or more) and other variables.

Variables	Categories	Adjusted Odds ratio	B	Confidence interval 95%		P-value
				Lower	Upper	
Gender	Male	Reference				< 0.001
	Female	5.6	1.7	3.226	9.387	
Total calcium (mg/dL)	8.6–10.2	Reference				0.738
	Below 8.5	1.42	0.35	0.183	11	
Creatinine (mg/dL)	0.7 to 1.3	Reference				0.4
	Above 1.3	1.1	0.3	0.7	1.9	
Phosphorus (mg/dL)	2.8–4.5	Reference				0.7
	Under 2.8	1.5	0.4	0.5	4.3	0.43
	Above 4.5	1.1	0.1	0.46	2.6	0.8
Aspirin usage	Yes	Reference				0.468
	NO	1.1	0.174	0.74	1.9	

#### After propensity matching score performance

**Figure 3:**
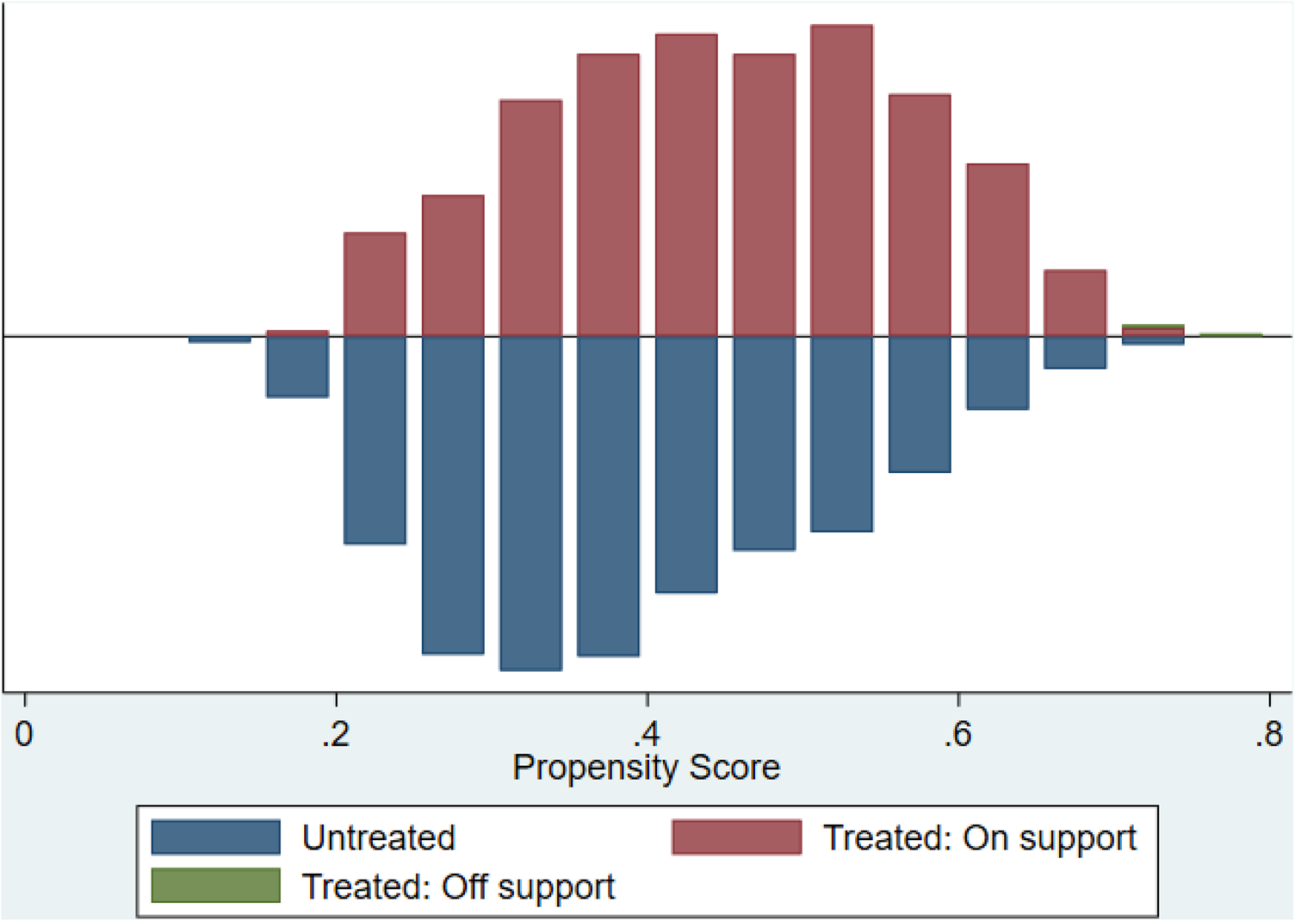
Graphical representation for the propensity matching score.

The model was adjusted for age, weight, height, gender, race, education, body mass index, family history of osteoporosis and history of severe trauma. Graphical representation for matching represented in Fig. 3. Logistic regression after matching did not show significant difference between risk for fractures and low aspirin dose (Table 5).

**Table 5:** Multiple Logistic regression after propensity Score matching.

Outcome	Adjusted odds ration	Low CI	High CI	P-value
Fracture wrist				
Aspirin use	0.84	0.64	1.09	0.19
Propensity score	1.54	0.55	4.36	0.41
Fracture spine				
Aspirin use	0.76	0.50	1.16	0.20
Propensity score	3.31	0.67	16.42	0.14
Fracture hip				
Aspirin use	0.90	0.56	1.44	0.65
Propensity score	2.21	0.35	14.05	0.40

## Discussion

Aspirin is a low-cost, safe anti-inflammatory drug which primarily has been used to treat and prevent cardiovascular diseases^24^. It is beneficial to bone regeneration by inhibiting osteoclastogenesis and facilitating osteogenesis and osteogenic differentiation^25^. This study, however, found that aspirin use among older adults had no impacts on the risk of bone fracture at hip, wrist, and spine, indicating that aspirin alone may not enhance bone health in this group of population. Our findings could be explained by the fact that the dosage of aspirin intake was not taken into consideration. There has been evidence showing that low and high dose of aspirin use may have different impacts on bone synthesis and degeneration, with low dose aspirin beneficial to bone synthesis but high dose may cause adverse effects due to its impact on both bone synthesis and degeneration^26^. Therefore, the amount of aspirin intake could be a limiting factor in the relationship with bone fractures. In addition, the duration of aspirin exposure could also influence the outcome. This factor, however, could not be considered in our study due to insufficient data.

Mixed evidence has been shown regarding the impacts of aspirin on risk of bone fracture. In a meta-analysis and review of 12 studies, a reduction of 17% odds of bone fracture was found when aspirin was administered^27^. However, most studies in this meta-analysis were cross-sectional study and none were randomized controlled trial, limiting the applicability of the findings. In a similar systematic review, it was concluded that although aspirin use has been associated with higher bone mineral density, its impacts on the risk of bone fracture has been inconclusive^28^, echoing the results of our study. A case control study, however, found an increase in risk of overall bone fracture and hip fracture with low dose of aspirin use^21^. It shows that the inconsistent relationship between aspirin use, and risk of bone fractures warrant further investigations.

Bone remodeling is a continuous process which involves bone resorption and bone synthesis^26^, and the balance of these determines the net gain or loss of bone mineral density. Although bone mineral density, an indicator of bone health, has been associated with aspirin use, the evidence has also been inconsistent in the last decades. Bonten et al.^19^ found that chronic use of low-dose aspirin was not associated with increased femoral and vertebral bone mineral density. In contrast, Carbone et al.^14^ found that aspirin use was associated with higher bone mineral density at the whole body, trabecular and spine. Bauer et al.^29^ consistently found that aspirin use was associated with 2–3–5.8% increase in bone mineral density at hip and spine among older women. There was, however, neither protective (Bauer et al.^29^) nor healing effect on bone fracture (Hunter et al. ^30^). In addition, the role of aspirin on bone mineral density is believed to be dose dependent. Low dose (< 100 μg/mL) aspirin use was found to facilitate bone formation and inhibit osteoclast activity, whilst high dose (150–300 μg/mL) aspirin use had double positive effects on both osteoclasts and osteoblasts activities, leading to conflicting evidence^21,26^.

There has been evidence showing that the incorporation of aspirin and other supplements could enhance bone regeneration. For example, the administration of aspirin together with β-tricalcium phosphate and poly-lactic-co-glycolic acid can lead to higher rate of bone formation than aspirin alone (Tao et al.^25^), indicating that only aspirin use may not optimize bone health. However, Chai et al.^31^ found that combination of aspirin and vitamin E could not prevent bone loss at whole body, femoral and lumbar. Further investigations are therefore warranted to assess the interactive effects of aspirin and other supplements.

The limitations of this study pertain to the cross-sectional setting in which causal relationship could not be drawn. In addition, self-reported data may be subjected to recall bias especially for this older group of population. However, this is a population-based study which has higher representativeness of the country, when comparing with other similar but smaller studies^14,19,29,30^. In addition, the duration of prophylactic aspirin use is not available on the NHANES database, and we were unable to incorporate FRAX and Garvan fracture risk calculators in the statistics since these variables were not uploaded to the NHANES database online within the timeline that we used for the research (2017–2020 and 2013–2014), in addition the minimum period on low aspirin necessary to be deemed exposed was not indicated on the Nhanes website. Therefore, the dose–response relationship between aspirin and bone fracture, and the relationship between the duration of exposure and the outcome, could not be identified. We faced a lot of missing data and unscreened patients, so we removed them to get the right and significant statistical outcomes related to aspirin usage and the fractures. Furthermore, we couldn't conduct a cross-sectional study of the last 5 years, from 2013 to 2020, due to lost data during 2015–2016. In addition, many variables should be extracted and then inserted into the statistical analysis, such as Albumin & Creatinine—Urine, CRP, and vitamin D level, to examine the most reliable interactions between all of them. In addition, we depended on the uploaded data on the NHANES database, which they concentrated on hip, wrist and spine fractures as well, as they are the most common fractures, thus we attempted to cover the study in the greatest category of fractures. Finally, we couldn’t perform multivariable logistic regression with adjusting BMD (after and before propensity matching score) due to the enormous quantity of missing data linked to BMD, and we have addressed this issue in the limitation section (Supplementary file).

## Conclusion

Many clinical studies attempted to explore the substantial effect of low-dose aspirin preventive use on the fractures in elderly individuals, including in vitro studies and animal experiments. However, our cross-sectional analysis of 7132 elderly persons from the NHANES database has confirmed the insufficient effect of low-dose aspirin preventive use on reducing the fractures, mainly due to a fall from a standing height or less, indicating osteoporosis. World health organizations should suggest many recommendations to stop the random use of aspirin in elderly persons due to the mistaken belief in its beneficial role in restoring bones, especially in countries that do not have strict control over the perception of medicines.

## Supplementary Information

Supplementary Information.

## Data Availability

The datasets generated and/or analysed during the current study are available in the National Health and Nutrition Examination Survey repository, [https://wwwn.cdc.gov/nchs/nhanes/continuousnhanes/default.aspx]. All data generated or analysed during this study are included in this published article [and its supplementary information files].
